# Clinical Presentations, Diagnosis, and Genetic Features of Hemophagocytic Lymphohistiocytosis: A Single Institutional Experience With the Saudi Population

**DOI:** 10.7759/cureus.61879

**Published:** 2024-06-07

**Authors:** Sami I Alradhi, Fahad Almanjomi, Fahad Alamr, Ibrahim Alwakid, Meshal Alrashidi, Mayada Alkhelaif

**Affiliations:** 1 Pediatric Hematology and Oncology, Maternity and Children Hospital, Dammam, SAU; 2 Pediatric Oncology, King Fahad Medical City, Riyadh, SAU; 3 College of Medicine, Al-Baha University, Al-Baha, SAU; 4 General Pediatrics, King Fahad Medical City, Riyadh, SAU

**Keywords:** unc13d, rab27a, prf1, stxbp2, saudi patients, hemophagocytic lymphohistiocytosis

## Abstract

Background

Hemophagocytic lymphohistiocytosis (HLH) is an uncommon, potentially fatal condition caused by high immune activation. The present study aimed to identify the clinical manifestations, geographic distribution, and associated pathogenic genetic mutations of HLH in Saudi Arabia.

Method

A retrospective cross-sectional study was conducted at King Fahad Medical City (KFMC), with a total of 59 patients diagnosed with HLH in the period between 2006 and 2018. All genetic results and clinical and biochemical data were retrieved and statistically analyzed using IBM SPSS Statistics for Windows, Version 25 (Released 2017; IBM Corp., Armonk, New York, United States).

Results

The results revealed that 48 patients (81.4%) had 15 pathogenic mutations of primary HLH whereas 8 (13.6%) patients had no genetic mutation. The most common variant mutation identified was c.1430C>T of the STXBP2 gene (42.4% of total patients), followed by c.1122G>A of the PRF1 gene (10.2% of patients), which demonstrated a distinctive geographic and tribal association. Patients with RAB27A mutation tend to present at an older age than the others with a median age of presentation of 5.5 months vs 2 months for patients with PRF1 mutations. No significant differences in clinical features were observed among the various groups.

Conclusion

This study highlights the incidence of genetic mutations among the Saudi population with HLH. The STXBP2 is the most common mutation followed by PRF1 mutations, many mutation variants are associated with a distinctive tribal and geographic association.

## Introduction

Hemophagocytic lymphohistiocytosis (HLH) is a rare and potentially fatal systemic inflammatory syndrome that primarily affects infants and young children. It was first reported in the literature in 1939 by Scott and Robb-Smith [1]. The disease was later recognized as a familial condition in 1952 by Farquhar and Claireaux [2]. HLH is characterized by the impaired cytotoxic function of natural killer (NK) cells which leads to a massive cytokine release, resulting in systemic inflammation. Consequently, this syndrome is manifested by fever, splenomegaly, pancytopenia, hypertriglyceridemia, hyperferritinemia, and hypofibrinogenemia related to uncontrolled and persistent T lymphocytes and macrophage activation [3].

Diagnosing HLH imposes a clinical challenge due to a lack of specific laboratory findings or pathognomonic clinical signs. HLH-2004 criteria from the Histiocyte Society are the most widely accepted diagnostic criteria for HLH [4]. It can be classified into two distinct forms such as primary HLH (pHLH), and secondary HLH (sHLH) [5,6]. The pHLH can be further classified into familial hemophagocytic lymphohistiocytosis (FHL), and primary immunodeficiency-associated HLH. The HLH-reported genetic mutation in PRF1, UNC13D, STX11, and STXBP2 further divides FHL into five subtypes (FHL1 to FHL5). pHLH can occur with primary immunodeficiency syndromes like Griscelli (RAB27A), Chédiak-Higashi (LYST), and X-linked lymphoproliferative syndromes (SH2D1A, and XIAP). This disease typically occurs in infancy, but it might also be found in older patients [7]. Furthermore, genetic defects can also be identified in asymptomatic individuals. According to Henter et al., up to 50% of patients diagnosed with HLH did not show any of this molecular mutation [5].

FHL demonstrates an autosomal recessive mode of inheritance, with a high incidence of parental consanguinity in FHL cases [8-10]. The incidence of FHL is estimated to be approximately 1 in 50,000 live births [11,12]. It has been noticed that FHL can be more frequent in some regions compared to others. For example, in Italy, the disease is three times more frequent in the southern regions than in the northern and central regions [13]. Similarly, the majority of FHL cases in Japan originate in Kyushu, the major southern island of Japan [12]. The fact that specific types of genetic mutations accumulate in limited areas is indicative of the founder effect of this disease. Several genetic defects responsible for the pathogenesis of FHL have been elucidated [14-19].

In Saudi Arabia, the FHL-5 subtype was reported initially in consanguineous families of Saudi Arabian and Turkish origin [16,17]. Subsequently, several Saudi patients from the same tribe have been found to have the same genetic mutation. We anticipate a higher incidence of the disease in Saudi Arabia due to its tribal nature and the high rate of consanguinity as compared to other regions.

## Materials and methods

Study design

This retrospective cross-sectional study was conducted at King Fahad Medical City (KFMC), a tertiary hospital in Riyadh, Saudi Arabia. The study enrolled pediatric patients below the age of 14 years who were diagnosed with HLH between January 2006 to December 2018. All patients in this study were screened before enrollment to ensure they met the histiocyte society HLH-2004 diagnostic criteria [4]. Informed consent was taken from all participants.

Data collection

The data was collected through a pre-designed Excel sheet from the patients' files who are treated at the Department of Pediatric Oncology. The collected data included information about the patient’s demographic characteristics, family history, lab data, molecular tests, and geographic distribution. Patients who met the HLH-2004 criteria were diagnosed with pHLH if they had a known pHLH genetic mutation, and/or early-onset disease (less than two years old) with a family history of a similar condition. Other patients without known genetic mutation who met the HLH-2004 criteria [4] were diagnosed as sHLH. Central nervous system (CNS) involvement was defined by the presence of one of the following: cerebrospinal fluid (CSF) pleocytosis, clinical abnormal neurological findings, and/or abnormal neuroimaging on computed tomography (CT) scan or magnetic resonance imaging (MRI).

Statistical analyses

All categorical variables such as gender, age group, clinical characteristics, and residence were represented using both numerical values and percentages. Continuous variables such as age were described using the median and interquartile range (IQR). To accommodate skewed data, non-parametric tests were utilized. Kolmogorov Smirnov test was used to check the assumption of normal distribution. For determining significant associations between categorical variables, the chi-square test or Fisher’s exact test was used based on the expected frequency in each cell. Fisher’s exact test was preferred for smaller frequencies (<5). A two-tailed p-value less than 0.05 was considered statistically significant. All data were entered and analyzed through the statistical package IBM SPSS Statistics for Windows, Version 25 (Released 2017; IBM Corp., Armonk, New York, United States).

Ethical considerations

Institutional research ethics board approval was obtained before conducting any study procedure and patients' identities were kept confidential. The study was reviewed and approved by KFMC’s International Review Board before the start of the study and the need for informed consent for the archival samples was waived (IRB Log Number: 19-617).

## Results

A total of 59 patients were enrolled in this study. Out of 59 patients, 56 (94.9%) patients were categorized as pHLH and 3 (5.1%) as sHLH. A total of 48 (85.7%) pHLH patients had a positive genetic mutation whereas 8 (14.3%) patients had a negative genetic mutation. Table 1 shows the age distribution of pHLH patients. The results indicated that patients with negative genetic mutations presented at an older age compared to those with positive genetic mutations. Forty-nine (87.5%) pHLH patients presented before one year of age 49 (87.5%) whereas 26 (46.4%) presented before the age of three months.

**Table 1:** pHLH age distribution pHLH: primary hemophagocytic lymphohistiocytosis

Age of presentation	Number of patients (%)	Positive genetic mutations					Negative genetic mutations (%)
		STXBP2 (%)	PRF1 (%)	RAB27A (%)	UNC13D (%)	Total (%)	
0 ≤ 3 m	26 (46.4)	14 (53.8)	6 (23.1)	2 (7.7)	1 (3.8)	23 (88.5)	3 (11.5)
3 m ≤ 1 y	23 (41.1)	14 (60.8)	4 (17.4)	2 (8.7)	2 (8.7)	22 (95.7)	1 (4.3)
>1 y	7 (12.5)	-	1 (14.3)	2 (28.6)	-	3 (42.9)	4 (57.1)
Total	56	28 (50.0)	11 (19.6)	6 (10.7)	3 (5.4)	48 (85.7)	8 (14.3)

Clinical and laboratory characteristics of the patients with pHLH at the time of diagnosis are provided in Table 2. A slight male-to-female predominant ratio was observed (31:25) in the study. A total of 51 (91.1%) patients were found to have a positive family history of similar conditions and/or consanguinity. This result was slightly higher in the patients with positive genetic mutation than in patients with no mutation (93.75% vs. 75%). CNS involvement was noticed in 55.4% of the patients. Patients with UNC13D and PRF1 had a higher rate of CNS involvement than the rest of the group (100% and 63.6%). Almost all patients were presented with fever (98.2%). Splenomegaly was also noticed in most of the patients (92.9%).

**Table 2:** pHLH clinical and laboratory characteristics CNS: central nervous system; pHLH: primary hemophagocytic lymphohistiocytosis

		STXBP2 (N = 28)	PRF1 (N = 11)	RAB27A (N = 6)	UNC13D (N = 3)	Negative genetic mutations (N = 8)	Total (N = 56)	P- value
Gender (M:F)		15:13	8:3	4:2	2:1	4:4	33:23	0.794
Consanguinity and/or family history		27 (96.4%)	9 (81.8%)	6 (100%)	3 (100%)	6 (75%)	51 (91.1%)	0.234
Median age (months)		2.5 (6-2)	2.0 (3-2)	5.5 (43.50-1.62)	4.0 (0-2)	8.0 (24.75-2.00)	3 (6.75-2)	0.282
Symptoms and labs								
Fever		27 (96.4%)	11 (100%)	6 (100%)	3 (100%)	8 (100%)	55 (98.2%)	0.907
Splenomegaly		26 (92.9%)	10 (90.9%)	6 (100%)	3 (100%)	7 (87.5%)	52 (92.9%)	0.894
CNS involvement		16 (57.1%)	7 (63.6%)	2 (33.3%)	3 (100%)	3 (37.5%)	31 (55.4%)	0.290
Neutrophils (<1.0x10^9^/L)		13 (46.4%)	7 (63.6%)	5 (83.3%)	3 (100%)	3 (37.5%)	31 (55.4%)	0.161
Hemoglobin (<90g/L)		24 (85.7%)	7 (63.6%)	4 (66.7%)	2 (66.7%)	6 (75%)	43 (76.8%)	0.583
Platelets (<100x10^9^/L)		26 (92.9%)	10 (90.9%)	5 (83.3%)	3 (100%)	5 (62.5%)	49 (87.5%)	0.203
Hypertriglyceridemia fasting (≥3.0mmol/L)		19 (67.9%)	4 (36.4%)	4 (66.7%)	3 (100%)	3 (37.5%)	33 (58.9%)	0.136
Hypofibrinogenemia (≤1.5g/L)		26 (92.9%)	9 (81.8%)	4 (66.7%)	3 (100%)	5 (62.5%)	47 (83.9%)	0.177
Hyperferritinemia (≥500µg/L) (total)		28 (100%)	10 (90.9%)	6 (100%)	3 (100%)	8 (100%)	55 (98.2%)	0.195
	500≤5000	12	5	3	2	1	23	
	5000≤10000	4	2	0	1	0	7	
	>10000	12	3	3	0	7	25	
Elevated liver enzymes		28 (100%)	8 (72.7%)	5 (83.3%)	2 (66.7%)	8 (100%)	51 (91.1%)	0.030
Hemophagocytosis		24 (85.8%)	8 (72.7%)	5 (83.3%)	3 (100%)	7 (87.5%)	47 (83.9%)	0.783

Genetic mutations in pHLH

After analyzing the results of 56 patients diagnosed with pHLH, 48 (85.7%) patients were found to have a positive genetic mutation. The results revealed a total of 15 pathogenic genetic mutation variants in four genes (STXBP2, PRF1, RAB27A, and UNC13D) (Table 3).

**Table 3:** pHLH associated pathogenic genetic mutations in 48 Saudi patients pHLH: primary hemophagocytic lymphohistiocytosis

Gene	Total number (%)	Nucleotide variant	N of patients
STXBP2	28 (58.3%)	c.1430C>T	25/28 (89.3%)
		c.703C>G	1
		c.1463C>T	1
		c.1485+1G>A	1
PRF1	11 (22.9%)	c.1122G>A	6/11 (54.5%)
		c.895C>T	2
		c.769T>C	1
		c.1168C>T	1
		c.658G>T	1
RAB27A	6 (12.5%)	Homozygous duplication of exons 2 to 5	2/6 (33.3%)
		c.395G>T (Heterozygous)	1
		c.148_149delinsC	1
		c.244C>T	1
		c.514C>T	1
UNC13D	3 (6.3%)	c.3048dup	3/3 (100%)

STXBP2

STXBP2 (FHL-5) was the most common genetic mutation identified in this study. Four pathogenic variants were found in a total of 28 (58.3%) patients (Table 3). A total of 25 (89.3%) patients were carrying the same homozygous mutation, c.1430C>T. All 25 patients were originally from Riyadh province whereas 18/25 (72%) patients belonged to the same tribe and the same town (Wadi ad-Dawasir). Another 5/25 (20%) patients shared the same tribe and were from the same geographic area (Al Duwadimi town and nearby villages). The other three mutations were c.703C>G, c.1463C>T, and c.1485+1G>A.

PRF1

Five pathogenic variants were isolated in FHL-2, the most frequent being c.1122G>A. It was isolated from six (54.5%) patients. Five patients with this mutation were originally from Jazan province (Table 3). Two patients (18.9%) from the same tribe and originating from Al-Jouf province were found to have c.895C>T mutation. The other three isolated mutations were c.769T>C, c.1168C>T, and c.658G>T.

RAB27A

We isolated five different variants of Griscelli syndrome type 2. The most prevalent variant was a homozygous tandem duplication of 38 kb affecting exon 2-5 and resulting in a premature stop codon. This variant was found in two patients originating from Al-Jouf province, both of them belonged to the same tribe. Although the mode of inheritance in this syndrome is autosomal recessive, a heterozygous c.395G>T mutation was found in one patient who was diagnosed clinically with Griscelli syndrome with pHLH. The other three isolated mutations were c.148_149delinsC, c.244C>T, and c.514C>T. Two (33.3%) patients with Griscelli syndrome type 2 presented at more than one year of age.

UNC13D

One pathogenic mutation variant was identified in three patients with FHL-3. All three patients with c.3048dup were from Al-Jouf province. As with all other pHLH, patients with UNC13D mutation had a high rate of a positive history of consanguinity and/or family history of similar conditions.

pHLH with negative genetic mutations and sHLH

These patients were diagnosed with pHLH either due to early onset of the disease, family history, consanguinity, and/or disease reactivation without clear cause like infection and autoimmune disease. A total of eight (14.3%) were diagnosed as pHLH without positive genetic mutations, four (50%) of them presented after the age of one year, and the other four (50%) patients presented earlier. No other significant clinical or laboratory characteristics differences were found in this group of patients. A total of three (5.1%) patients were diagnosed with sHLH. Two patients were diagnosed with primary immune deficiency syndromes, and the third patient was diagnosed with rheumatological disease.

Associated infections with HLH

STXBP1

Out of 28 patients with STXBP1, 9 (32.14%) patients were found to have associated infections. Cytomegalovirus (CMV) virus was detected in two patients. Furthermore, a respiratory panel swab showed two patients with rhinovirus and one with coronavirus. Two patients were found to have positive blood cultures, one with *Staphylococcus epidermidis* and the other one with *Streptococcus viridans*. *Klebsiella pneumonia* was also isolated from one patient’s urine culture. The last patient was found to have *Clostridium difficile* in the stool.

PRF1

Five out of 11 patients with PRF1 gene mutation were found to have associated infections. CMV virus was detected in two patients. Both of them also had co-infections, with one having *Pseudomonas aeruginosa* in the blood and the other one having herpes simplex virus (HSV) in the blood. *Escherichia coli* and *K. pneumonia *were positive in the urine culture of one patient. Respiratory syncytial virus (RSV) was positive in one patient respiratory panel swab. CSF virology study was positive for HSV in one patient.

RAP27A

Among patients with RAP27A mutation, rhinovirus was isolated in the respiratory panel swab of one patient. Another patient was found to have Epstein-Barr virus (EBV) in the blood.

UNC13D

Patients with UNC13D were also identified to have infections. The blood culture of one patient revealed *E. coli *whereas another patient was found to have CMV in the blood.

pHLH with negative genetic mutations and sHLH

Six out of eight (75%) patients with no identified gene mutation were found to have associated infections. Three patients were CMV positive, and two of them were co-infected with *E. coli* in one patient and *Staphylococcus hominis* and EBV in the other patient. Two patients tested positive for EBV. They were co-infection with *Pseudomonas putida* in the blood of one patient and rhinovirus was found in the respiratory panel of one patient. Blood culture was positive for *E. coli* in one patient whereas another patient was found to have EBV in the blood.

## Discussion

Familial HLH is a very rare disease in the world; however, it may not be that uncommon in Saudi Arabia, given the tribal nature and high rate of consanguineous marriages. This is evidenced in the present study in which 91.1% of patients had a family history of consanguinity and/or a strong family history of similar conditions. Our findings showed that 94.9% were suffering from pHLH. Most cases of pHLH are diagnosed during the first year of life with a median age of onset of 5.1 to 9.5 months which is comparable to the present study (three months) [20-22].

The average survival time after the diagnosis of HLH is less than two months if left untreated [2]. This, when coupled with the fact that the diagnosis of HLH is often delayed, compounds the risks for patient outcomes. For example, severe fever in HLH is often mistaken for sepsis, resulting in delays in diagnosis. Tseng et al. and Cleves et al., in their studies, reported a median duration of 34.5 and 17 days, respectively for the diagnosis of HLH [23,24]. The identification of Saudi HLH patients’ specific characteristics reported in this study will allow early recognition and a prompt introduction of immunotherapy and/or stem cell transplantation which can significantly improve overall survival [25].

In our study, the main symptoms at initial presentation included fever (98.2%), splenomegaly (92.9%), and cytopenia which aligns with the existing literature [26]. Fever is one of the eight conditions that are part of the revised 2004 guidelines for HLH diagnosis and has been reported in more than 90% of the cases [4,27]. Other clinical findings include elevated liver enzymes (91.1%) which is a common finding in HLH. Some authors suggest searching for another diagnosis in the case of normal liver enzymes [28-30]. In the literature, CNS involvement was observed in 30% to 73% of HLH patients, and elevated ferritin is observed in 97%, which is comparable to our results of 55.4% and 98.2%, respectively [31,32].

International studies showed that mutations of PRF1, UNC13D, and STX11 are the most commonly isolated ones in patients with pHLH. However, our findings showed that the most common genetic mutation in Saudi Arabia is STXBP2 [33-36]. c.1430C>T was observed in 89.3% of our patients with STXBP2, the index cases of these mutations are from the Arabian Peninsula which was first reported by Côte et al. [19]. The majority of the patients (89.3%) with this gene mutation were originally from Riyadh province. Five out of six patients with mutation variant (c.1122G>A) of PRF1 are originally from Jazan province. Furthermore, all three patients with mutation (c.3048dup) variant of UNC13D and two patients with (homozygous duplication encompassing exon 2 to 5) RAB27A are from Al Jouf province. These are significant findings as this has never been reported in Saudi Arabia.

Other undetected types of pathogenic variants may have also been present; however, further research is required in this regard. In this study, no genetic mutations were detected in eight (14.3%) patients although six of them presented with a strong history of consanguinity and/or similar conditions in the family. Furthermore, seven patients presented before the age of two years. It has been reported in the literature that up to 50% of patients with HLH present with no obvious genetic mutations [4,37]. These eight patients might have a novel mutation that has not been discovered yet as a cause of pHLH.

Several etiological factors can be responsible for HLH, including various infections which may present a wide range of clinical manifestations and lead to a high rate of morbidity and mortality [38]. The most common trigger of pHLH in this study was found to be viral infections. CMV, EBV, rhinovirus, RSV, HSV, and coronavirus were all been isolated in our patients. The most common viral infection identified in the present study was CMV. However, this does not align with the literature as the most common viral agent described in the literature is EBV [39]. For example, Cleves et al. in their study reported that almost 66% of their studied population had viral etiology, with 52.3% having EBV infection [24]. However, CMV has also been reported in various viral HLH cases [40,41]. Bonnecaze et al. reported a confirmed case of HLH caused by acute CMV in an immunocompetent host [40]. The bacterial infections also were isolated in many patients either from blood or urine. All organisms isolated were reported previously in the literature [42-45].

There are several strengths and limitations of the study as well which should be considered while interpreting the findings. The main strength of the study is retrospectively analyzing the data over a period of 13 years. Furthermore, Saudi Arabia is a relatively unexplored region regarding HLH. The reporting of genetic mutations from the region could help future studies relate and build on these findings to further elucidate the mechanism of HLH and its management. There are some limitations of the study as well. The main limitation of the study is the small sample size. This could be because HLH is quite a rare disease and the study was only conducted at a single tertiary hospital. Future studies should focus on larger patient populations and have a longer follow-up period.

## Conclusions

It has been known that HLH is a rare disease worldwide, but this could not be the case in Saudi Arabia due to its higher rate of consanguinity and its tribal nature. Based on our data the incidence of HLH was higher in both gender and different ages. Also, our results show the most common mutated genes in the Saudi population and this data might be considered representative of Saudi as it was conducted in a tertiary hospital where there is a high number of referral cases from all over the kingdom along with a patient referral from the Riyadh city. Early recognition and prompt introduction of treatment will significantly improve the overall survival of the patients. Further researches are required to consolidate what has been demonstrated in this study.
